# Ethnobotanical study of wild edible plants in Derashe and Kucha Districts, South Ethiopia

**DOI:** 10.1186/1746-4269-2-53

**Published:** 2006-12-21

**Authors:** Kebu Balemie, Fassil Kebebew

**Affiliations:** 1 Department of Ethnobiology, Institute of Biodiversity Conservation, Addis Ababa P.O. Box 30726, Ethiopia

## Abstract

The study discussed ethnobotany of and threats to wild edible plants in Derashe and Kucha Districts, South Ethiopia. Semi-structured interview, field observation, group discussion, market survey, and pair wise ranking were employed to gather ethnobotanical data. The information was collected from informants of three ethnic groups namely, Kusume, Derashe and Gamo people. The study documented 66 edible plant species belonging to 54 genera and 34 families. Of the reported edibles, 83.3% have more than one use categories. Food, medicine, construction/technology, and fuel wood had contributed 79% of the total uses. Of the recorded wild edible plant species, 78.8% were reported to be edible both in normal and food shortage times. Procurement and use of most edibles were found to be age and gender specific. However, species use under various use categories does not vary among the communities (X^2 ^= 3.89, df = 6, α = 0.05 and 1-α = 12.6). The study showed that the majority (62.1%) of the species were collected from wooded grassland/or bush land vegetation type. Pair wise ranking results indicated that agricultural expansion, over stocking/overgrazing, fuel wood collection, and uncontrolled fire setting as principal threats to wild edible plants in the study areas. The findings suggest that (i) Public awareness and community based management need to be encouraged at all levels in order to overcome the threats; (ii) further investigation into nutritional properties of all the species reported; and (iii) Since the species are also nutraceutical, study on the pharmacological attributes would help to understand their medicinal applications. Furthermore, urgent collection of germplasm from areas under human pressure is recommended.

## Background

Millions of people in many developing countries do not have enough food to meet their daily requirements and a further more people are deficient in one or more micronutrients [1]. Thus, in most cases rural communities depend on wild resources including wild edible plants to meet their food needs in periods of food crisis. The diversity in wild species offers variety in family diet and contributes to household food security. Numerous publications provide detailed knowledge of edible wild plants in specific locations in Africa [e.g. [2,3] and [4]]. All showed that wild plants are essential components of many Africans' diets, especially in periods of seasonal food shortage. A study conducted in Zimbabwe revealed that some poor households rely on wild fruits as an alternative to cultivated food for a quarter of all dry season's meals [5]. Similarly, in Northern Nigeria, leafy vegetables and other bush foods are collected as daily supplements to relishes and soups [6]. In Swaziland, wild plants is still of great importance and contribute a greater share to the annual diet than domesticated crops [7]. Various reports also noted that many wild edibles are nutritionally rich [7-9] and can supplement nutritional requirements, especially vitamins and micronutrients. Nutritional analysis of some wild food plants demonstrates that in many cases the nutritional quality of wild plants is comparable and in some cases even superior to domesticated varieties [10].

Earlier works showed that about 8% of the nearly 7000 higher plants of Ethiopia are edible. Of these, 203 wild and semi-wild plant species are documented [11]. Still many more wild species are believed to be edible and undocumented yet. More recently, some ethnobotanical studies have undertaken in some parts of the country. However, the majority of these studies have dealt with medicinal species and little emphasis has been paid to wild edible plants. This study has therefore sought to document indigenous knowledge related to uses of wild edible plant species and to assess the existing threats to wild edible plants in the study areas.

## Materials and methods

### Study area and people

**Figure 1:**
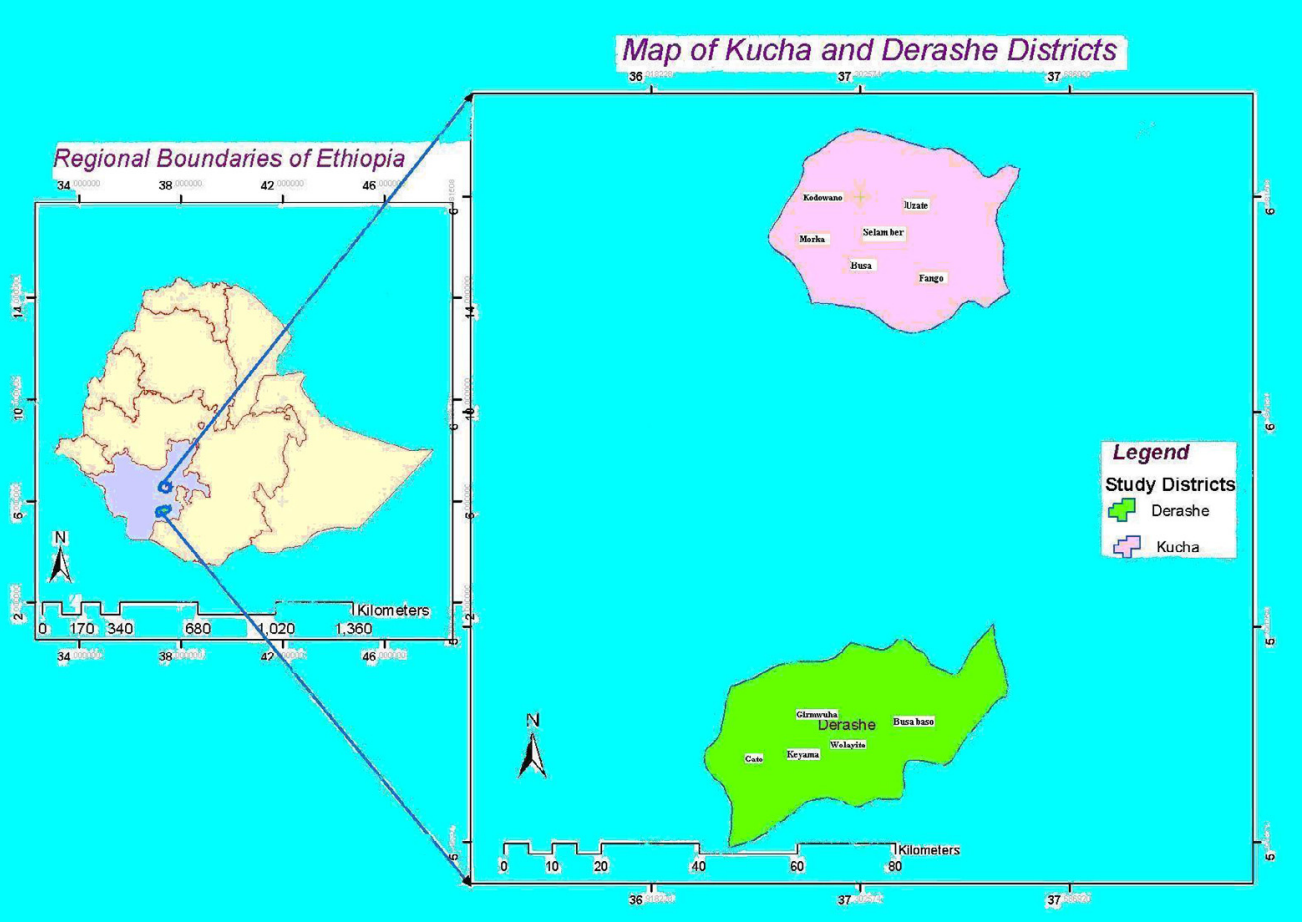
Map of Ethiopia showing study areas (Derashe and Kucha Districts).

The study was conducted in Derashe and Kucha districts, South Region of Ethiopia with three ethnic groups namely, Kusume, Derashe and Gamo communities (see Figure 1). The Kusume people (population about 8,543) live in low land areas of Gato (altitude <1300 m). The Derashe people (population 91, 654) live in the low and high land parts (1250–2300 m) of Derashe district. The Gamo people (population about 126,561) live in a more isolated district of varying topography ranging between (1250–2600 m) [12]. The Derashe and Gamo are the dominant ethnic communities of Derahse and Kucha districts respectively. In all communities, the majority of the people practice similar economic activities, mixed farming (crop and livestock production). Of the three communities, the Kusume and Derashe share overlapping ecological niches, culture and languages. The districts cover humid highland (>2000 m), intermediate climate and semiarid lowland (<1500 m.). The vegetation cover of the study areas varies from patches of tall forest trees on the slop of the escarpments to wooded grassland of the plain areas of both Derashe and Kucha districts. The low land areas are covered by various shrubs and savannah grasses in Kucha district. Some of the common shrubs of bush land and wooded grassland include small-leaved species of *Acacia, Commiphora *and broad-leaved species of *Combretum *and *Terminalia*. On the hills and hill slopes, tree species like *Juniperous procera*, *Podocarpus falcatus*, *Syzygium guineense *and *Olea *spp *europaea *occurred here and there.

### Methods

Five study sites from Derashe district and six from Kucha were conveniently selected based on vegetation cover and altitudes. Seventy-two informants of different age groups (35 from Gamo, 19 from Derashe and 18 from Kusume) were interviewed. The informants were selected with assistance of agricultural experts and Development Agents (DA) in study areas. The study was conducted using semi-structured interview, field observation, group discussion, market survey and pair wise ranking. To understand local peoples' perception on activities threatening wild edible plants, pair wise ranking [13] was conducted and the number of possible pairs was calculated using the relation N (N-1)/2, where N is the number of factors (activities). Accordingly, five factors threatening wild edible plants were identified with the community. The total number of pairs was determined using the formula, and the ten pairs arranged and presented to informants to choose one from the two threats at a time. Then, the scores from each respondent summed-up, the ranks determined and the factor that received the highest total score ranked first. Specimens of wild edible plants were collected, identified, and deposited at the National Herbarium, Addis Ababa University. Information gathered through semi-structured interview was presented using percentages and ranking. Chi-square (X^2^) statistical test of homogeneity was calculated to test the null hypothesis that states, "There is no difference in use of wild edible plants under various use categories among the study communities". Furthermore, correspondence analysis was exercised for use report frequency and the first six axes, which contributed 79% of the total variation, were used as input for cluster analysis. Ward Square Euclidean distance in MINITB computer software was applied to investigate species use similarity [14].

## Results and discussion

### Taxonomic diversity

The flora of the study area is rich and provides diverse useful species. The study documented 66 wild edible plant species classified among 54 genera and 34 families. List of all the recorded plant species and uses are presented (see Additional file 7). Anacardiaceae and Tiliaceae families had the highest proportion of edibles with 5 species each. The results of the study revealed that the majority (83.3%) of the species have multiple uses and serve for more than one use categories. Four major use categories namely, food, fuel wood, construction/technology, and herbal medicine had contributed 79% of the total uses. Most of the recorded and identified species are reported to be edible elsewhere in Ethiopia and in other countries of Africa. For example, of the species recorded in this study, some authors [3,15] documented 39 and 14 species as edibles respectively. The growth forms of the species include shrubs, trees, herbs and climbers. Shrubs and trees make up the highest proportion of the edible species. Fruits, leaves, and seeds are the parts used widely by the three communities in the study area. The results also revealed that some of the reported species are familiar and used commonly in the study areas (Table 1).

**Table 1:** Wild edibles species with high use report and their altitudinal distribution

Scientific Names	Parts Used	Use Report (%)	Altitudinal distribution
*Amaranthus caudatus*	Young leaves	46.60	1250–2300 m
*Balanites aegyptiaca*	Leaves & fruits	100.00	1250–1300 m
*Carisa spinarum*	Fruits	100.00	1300–2000 m
*Corchorus olitorius*	Young leaves	63.01	1300–2300 m
*Corchorus trilocularis*	Young leaves	42.47	1300–2300 m
*Cordia africana*	Fruits	100.00	1300–1800 m
*Ehretia cymosa*	Fruits	47.95	1500–2000 m
*Ficus sycomorus*	Fruits	100.00	1300–1650 m
*Ficus vallis*	Fruits	100.00	1300–1650 m
*Portulaca quadrifida*	Young leaves	100.00	1200–1600 m
*Sclerocarya birrea*	Fruits	100.00	1200–1500 m
*Solanum nigrum*	Fruits	79.45	1300–2000 m
*Syzygium guineense*	Fruits	90.41	1500–1800 m
*Zanthoxylum chalybeum*	Fruits	38.36	1300–1700 m
*Ximenia *a*mericana*	Fruits	100.00	1250–1500 m

### Local knowledge and use

The Kusume people affirmed that 35 (53%), Derashe 36 (54.5%) and Gamo 40 (60.6%) of the 66 edibles recorded in this study are edible species. Thirty-one species are shared and commonly used among the communities in the areas at different levels. Edibility of these species over wider areas among different communities indicates the existence of common knowledge across a range of subsistence groups of different culture and geographic areas. In comparison, the Kusume and Derashe shared more number of edible species. Sixteen species have even similar names in Derashe and Kusume languages. Similarity in knowledge of these species is also reflected in the preparation of the traditional food known as *kurkufa*, a cultural dish prepared usually from maize or sorghum flour cooked with leafy vegetables. The species use similarity between Kusume and Derashe communities may be attributable to the sharing of an overlapping ecological niches, culture and languages. Interaction between communities through trade or due to proximity with one another might have over the years passed on the culture and knowledge on use of certain edible species to others [16]. Nevertheless, the sharing of overlapping ecological niches by the Kusume and Derashe people might some times compel the two communities to compete for the dwindling wild edible plant resources in their overlapping ecological niches. It is worthwhile to mention that *Cordeauxia edulis *is threatened because of over use in times of crop shortages in Southeastern Ethiopia [11]. The Gamo people, on the other hand, inhabits a more isolated district and has no ecological niche overlap with the other two ethnic groups. Thus, such competition could not exist.

Regarding collection and use of the edible plants, most of the edible fruits and seeds are collected and immediately used by children. Such foraging activities provide essential supplies of vitamins and minerals particularly to children [5,8,11,17]. On the other hand, collection and preparation of leafy edibles such as *Corchorus olitorius, Amaranthus caudatus *and *Portulaca quadrifida *are limited to women and young girls. The dishes prepared from these leafy edibles are however, consumed by all groups of the population. However, the local people appreciate some edible plants over the other in their utilization. For example, in Kusume areas, after *Moringa stenopetala*, *Leptadenia hastata *(*Hayla *in Kusume language) is preferred over the other leafy edibles. Similarly, in Gamo areas, *Solanum macrocarpon *(*Bulo *in Gamo language) is commonly used next to *Moringa stenopetala*. The informants asserted that *Moringa stenopetala *and *Solanum macrocarpon *are edible and medicinal species used in most areas of the South Region of the country. Similarly, some edible fruit bearing species such as *Ficus sycomorus, Sclerocarya birrea, Syzygium guineesne *and *Ximenia *a*mericana *are highly prized by individuals in all age groups. The reasons for appreciation of one species over the other, as said by most informants were easiness to process, nutritional value and taste during consumption.

The time and frequency of harvesting varies from plant to plant depending on the availability of edible plants/parts, which in turn vary from place to place due to ecological and climatic conditions. For example, *Syzygium guineesne *and *Ximenia americana *produce edible parts between March and April and are best collected for consumption within 1–2 months time. On the other hand, some weedy vegetables such as *Corchorus olitorius, C. trilocularis, Amaranthus caudatus, A. graecizans *and *Portulaca quadrifida *are available only on seasonal basis. In other words, they are available mainly during the rainy season (mainly between July and September) but harvesting depends on availability of food in stock. Some times these weedy species are also available in irrigated fields even during dry season.

### Local dishes

The edible parts are used as cabbage, fresh fruit or fruit juice, hot drink, boiled or roasted grain and tuber. Analysis of the results indicates that nearly 85% of the recorded edible species or their parts are consumed fresh with out further processing and most of them are fruits and seeds. Fruits of some species (e.g. *Syzygium guineesne, Ximenia americana*) are also used to make juice. Very few species are used as roasted/boiled grain or as hot drinks (as coffee and tea substitutes). For example, leaves of *Lanatana rhodesiensis *are roasted on iron plate; pounded, and then boiled in clay pot for use as hot tea to reduce the feeling of starvation. Most of the leafy edibles are however, consumed after being prepared as *kurkufa*. In preparing *kurkufa*, small boluses, which are made from unleavened dough of maize, sorghum, or barely are added into clay pot/jar containing boiled leafy vegetables and leftover on fire; when the mixture matures, excess water is drained off; salted and mixed with butter to improve the flavor. The *kurkufa *is then put onto *toma*, local bowl made usually from *Ficus *spp. and served along with unleavened (*kita*) or leavened bread. Additionally, the leafy edibles are used in preparing local nutritious drink known as *cheka *in Kusume area. In processing *cheka*, boluses are made from milled leafy edibles mixed with grains such as maize, sorghum or barley and added into clay jar containing boiled water and leftover fire. The cooked boluses are picked out of the jar and further enriched with additional flour of malted maize or barley and is kept for overnight in wooden bowl. The next morning, it is poured into clay pot/jar and left for a day; and then warmed mixture of either maize or sorghum flour is added to it; flour from brewed barley or maize is again added to it and left for overnight after which *cheka *is mixed with cold or warm water as required and served.

### Socio-economic significance

In addition to food value, the identified species are marketable and provide the opportunity to supplement household income. A study has shown that lower returns from farm necessitate the diversification of incomes from the sale of wild resources [8]. This is truly observed in the study areas where various wild edible plants were sold at local market (see Additional files 1 and 2). Of the recorded species, 12% are marketed as edible fruit or leaves; 7.6 % traded for timber and 49.3% for firewood at local market. Besides, *Moringa stenopetala *and *Solanum macrocarpon*, which are commonly marketed, some wild edibles such as *Balanites aegyptiaca, Opuntia ficus-indica, Leptadenia hastata, Ximenia americana*, and *Sclerocarya birrea *are also occasionally marketed at *Gato *market place by children and some Kusume women. Other economically important and marketable species is *Cordia africana*. It is the most preferred timber species and fetch high price at local market. In general, income derived from the sale of wild plant species is of particular importance to the poorer households who must supplement food production with cash in order to meet basic needs.

### Famine wild edibles and associated problems

Most of the wild edible plants recorded in this study are edible both in normal times and during food shortage. However, some (27.3%) wild edible plants are consumed only during famine or in times of food shortages. Famine foods are used only when preferred alternatives are not available and in situations where chronic food shortages prevail. On the other hand, although most of the famine edible species are useful in periods of food shortage, some of them contain substances that incite harmful reaction resulting in illness when ingested by humans or animals. In Kusume and Derashe areas, for example, informants reported that *Amaranthes graecizans *and *Portulaca quadrifida *cause anemia and body weakness when consumed in larger quantity and/or for extended period. Similarly, *Corchorus olitorius *cause dysentery when consumed continuously. Moreover, Kusume informants claimed that fruits of *Balanites aegyptiaca *incite burning sensation in stomach when consumed in larger quantities and/or extended period. Leaves of *Pentarrhinum inspidum *are fatal when fed to cattle. In Gamo areas, goats die when browsing on fruits of *Lepisanthes senegalensis*. Other famine edible species are either laborious to harvest or take long processing time before use. For example, cooking fruits of *Dobera glabra *takes the whole day. Likewise, digging soil and gathering roots of *Dioscorea praehensilis *is a difficult and laborious job.

Table 2 presents the more prominent edible species consumed during famine in the study areas. Most of these famine edible plants are weedy and often used as leafy vegetables during food shortages. Consumption of vegetal matter from wild plants has more regularity and higher intake proportion in times of food shortages [11]. The edibility information of these species was largely obtained from the low-income group. Nevertheless, with the advent of relief aid through food for work program, the use of some famine wild edible plants (e.g. *Sporobolus pyramidalis *and *Dioscorea praehensilis*) is diminishing. Similar study also showed that *Sisymbrium officinale *appears to have gradually lost its importance due to changes in food habits that favored the use of other varieties [11]. The reduced utilization of these species may gradually lead to the fading away of indigenous knowledge associated with the species and thus poses danger on poorer people who have relied on these cheap and relatively easily accessible food plants. Hence, efforts should be made to promote the uses of these wild edibles through genetic and nutritional studies, and developing appropriate processing methods. To this end, ethnobotanical knowledge is an important entry point and basic pragmatic information for further research on and development of these wild edibles.

**Table 2:** List of some commonly used famine edible plants

Scientific name	Local name	Parts used	Habit	Ethnic group
*Amaranthus graecizans*	Raza/horsokota, cumadhe	Leaves	Herb	All
*Amaranthus caudatus*	Gegebsa	Leaves	Herb	Gamo
*Commelina diffusa*	Welilo	Leaves	Herb	Gamo
*Corchorus olitoriu*	Hololoka/kefotugunta	Leaves	Herb	All
*Corchorus trilocularis*	Shosha interse	Leaves	Herb	Gamo
*Dioscorea praehensilis*	Welo	Root	Climber	Gamo
*Dobera glabra*	Kerseta	Seed	Tree	Kusume
*Hypoestes forskaolii*	Ononayita	Leaves	Herb	Kusume
*Leptadenia hastata*	Haila	Leaves	Climber	Kusume
*Portulaca quadrifida*	Merekita/meredeta/mergude	Leaves & stem	Herb	All
*Sporobolus pyramidalis*	Girole	Seed	Herb	Gamo

It is interesting to mention that some famine edible species of *Amaranthus, Corchorus *and *Portulaca *are domesticated and used widely as traditional leafy vegetable in most African countries such as Uganda, Kenya, Ghana, Nigeria, Zambia, South Africa, Botswana, and Tanzania [18]. For example, in Kenya, about 200 plant species, most of which wild, are consumed as leafy vegetables [16]. Similarly, in Mediterranean countries, leafy vegetables are regularly gathered from the wild to add varieties as well as nutrition to their diets [19]. A study conducted in Northern Senegal also showed that wild foods are commonly used to meet seasonal shortage of vitamins, which occur at the beginning of the wet season [20]. Works of various authors [3,5-7] have also shown the importance and critical roles of wild plants in supplying seasonal food needs and maintaining nutritional quality of traditional diets. Nutritional analysis of some leafy edible plants further showed that they have even better food values than cultivated vegetables [10]. In summary, therefore, the potential of wild food plants species in food and nutritional security, health and income generation has been increasing in the face of the growing environmental and socio-economic changes [18].

### Use diversity and habitat distribution

Analysis of use diversity showed that the recorded edibles species provide13 different uses to local communities. About 83.3% of the species are used for more than one use categories. Although some species have multiple uses, the average number of uses per species is three. The uses were placed under four major use categories, which had highest (79%) contribution of the total uses. These include food, medicinal, fuel wood, and construction/technology. Minor uses were categorized as miscellaneous since their contribution to the total uses is very small compared to others. Percentage of general utility of the plants among the study communities was evaluated using Chi-square (X^2^) test of homogeneity. The X^2 ^value (X^2 ^= 3.89; df = 6; α = 0.05 and 1-α = 12.6) indicated that the number of species reported as useful by the three communities under various use categories does not vary greatly implying that these uses are the common services obtained from wild edible plants in the study areas (Table 3).

**Table 3:** Comparison of percentage of general utility of wild edible plants among the study communities (Kusume, Gamo, and Derashe)

Ethnic Groups	Edible	Medicinal	Fire wood	Construction & Technology	Row Total	Chi-Square
Kusume	53.0	7.5	22.7	27.3	110.5	X^2 ^= 3.89 ns
Derashe	54.6	12.1	19.7	28.8	115.2	
Gamo	60.6	10.6	28.8	47.0	147.0	
Column total	168.5	30.2	71.2	103.1	372.7	

ns: no significant difference

**Figure 2:**
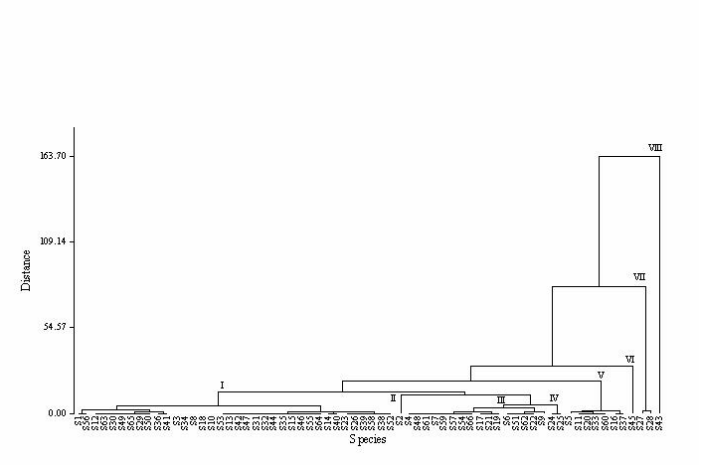
Dendrogram showing species use similarity.

Furthermore, species use similarity was analyzed from use report frequency. The dendrogram separated and grouped the 66 species into eight clusters (see Figure 2). Cluster I, which is an aggregation of 36 species, is separated from the rest and includes species reported for fuel wood and forage use values. Cluster IV, VI, VII and VIII are represented by 2, 7, 1, 2 and 1 species each, respectively. The species reported under these clusters have minor uses such as beehive hanging, rope making, walking stick and basket making. Cluster II and III include edible species reported for medicinal and construction values respectively. Cluster V includes species that have environmental (shade) and cultural (rituals) importance. Species such as *Celtis africana, Cordia african *and *Tamarindus indica *were reported to have both shade and ritual use values. Generally, the clusters indicate greater similarity, which might attribute to the similarity in uses of the species under various use categories.

Apart from the use diversity, the habitat distribution of the surveyed wild edibles was found diverse ranging from low to high land (1250–2300 masl.). The vegetation formation or habitat types of these wild edibles were forest, wooded grass/bush land, riverbanks, and farmland/abandoned field. The study revealed that most (62.1%) of the edible species were collected from lowland wooded grassland or bush land (see Additional file 7). These species were mainly fruit and seed bearing plants. Other edible species recorded in this study were distributed in farm field/farm border, and abandoned fields. Most of these species are weedy and have a broad range of altitudinal distribution (1250–2300 masl.). They become abundant after short rain. Their ability to grow fast and harvestable within short periods makes them useful in sustaining nutritional requirements in periods of food shortage.

In addition to the wild edibles, some economically useful plants, which are domesticated but still found in the wild, were encountered in Kucha District. These include *Coffee arabica, Rhamnus prinoides *and *Chata edulis*. The distribution of these species in the wild is narrowly restricted to far distant patchy forests. Similarly,*Capsicum annuum*, which is cultivated in some parts of the country, was found in the wild around riverbanks. The occurrence of this species in the wild might be through various dispersal mechanisms and perhaps a kind of escape from cultivation.

### Threats to and conservation status

Wild edible plants are facing threats in their natural habitats from various human activities. The level of impacts of these activities varies from place to place. To understand local people's perception on the activities/factors more threatening wild edible plant species, pair wise ranking of five factors (overstocking/over grazing, selective cutting for construction and technology, agricultural land expansion, fuel wood collection and uncontrolled fire setting) were conducted. Ten possible pairs were obtained from N (N-1)/2 relations for pair wise ranking, where N is the number of factors.

The total sum of each factor varies among informants of different communities (Table 4). The Derashe and Gamo informants rated agricultural expansion as the principal threat to wild plant species. This is mainly due to increasing demand for arable land by the burgeoning human population. Other activities ranked second with were overstocking and fuel wood collection in Derashe areas. Informants from Kusume have also reported over stocking/grazing followed by fuel wood collection and agricultural expansion as important factors affecting wild edible plants negatively. The reduction of grazing land due to agricultural expansion has possibly resulted in overstocking in this area. Similarly, the Gamo informants claimed fire wood collection as equally important factor as agricultural expansion in threatening wild plants including edible species. Uncontrolled fire setting was another important threat to wild plants in Kucha District. Interview responses indicate the local community traditionally used to practice fire setting to enhance growth of tender grass; control tsetse fly, ticks, and snakes. However, nowadays, some individuals set fire to expand agricultural land. It was observed that many woody species were severely affected by such fires where the tree and shrub stands declined (see Additional files 3 and 4), some even completely burned, others dried and collected as firewood, even the newly grown vegetative parts of woody species were further over browsed and trampled by livestock causing considerable damage to the species. A study conducted in Miombo Woodland, Tanzania has shown that fire affects many woody species including fire tolerant species when the duration of fire is long enough [21]. Furthermore, fuel wood collection is another problem facing wild plant species in the study areas. Many woody species are marketable at towns in Derashe district. Selective harvesting of some exceptionally useful plant species has also caused the depletion of the species. For example, species like *Cordia africana *and *Bridelia micrantha *are widely harvested for timber and walling or poles respectively (see Additional files 5 and 6). The occurrence of these species in the area was very low indicating the over exploitation pressure on the species. These two species are endangered and among the top ten considered for conservation in southwest Ethiopia [22].

**Table 4:** Results of pair wise ranking of factors considered as threats to wild edible plants

**Factors**	**Respondents**												**Total**	**Rank**
			
	*D1	**D2**	**D3**	**D4**	*G1	**G2**	**G3**	**G4**	*K1	**K2**	**K3**	**K4**		
			
Agricultural expansion	3	2	3	3	4	2	2	4	1	3	2	3	32	1
Fire	1	2	1	3	3	1	4	3	2	1	1	2	24	4
Fuel wood collection	3	2	3	2	2	3	2	1	3	3	2	2	28	3
Over-stocking/grazing	2	4	3	2	1	4	1	1	3	2	4	3	30	2
Selective harvesting	1	3	2	0	4	1	3	2	1	1	0	1	19	5

NB :-*(D = Derashe; G = Gamo; K = Kusume key informants). The scores in the Table are the value obtained from 12 key informants at four sub sites from each community.

As to the conservation status, most of the wild species in the areas have no protection. Especially the low land vegetation, which is the potential source of wild edibles, is now shrinking. Nevertheless, very few economic tree species (e.g. *Cordia africana*) are now managed by some farmers in their farmland as agroforestry tree and/or garden tree. This shows that such management of, and acquisition of economic benefits from species might promote local peoples' interest in conservation and maintenance of such locally important and endangered species.

## Conclusion

The result of the study revealed that knowledge about the edibility, habitat distribution, harvesting time and uses of most wild edible plant species is still maintained among the study communities. The preservation of this knowledge appears to be the result of continued reliance of local communities on the wild edible plants. Analysis of the results showed that in all study communities, most of the edible plants are used mainly by children and poor families both during normal and difficult times. Utility of the wild edible plants especially by younger community members ensure the maintenance of indigenous knowledge associated with the species. However, the decline in use of some famine edible species may gradually lead to the fading away of the indigenous knowledge associated with the plants. The results also revealed that many wild species are under growing pressures from various anthropogenic factors. Thus, public awareness and community based management need to be encouraged at all levels alongside of urgent collection of germplasm. The findings suggest further investigation into nutritional profiles and processing methods of all the species reported and study of the pharmacological properties for the nutraceutical species since they are also used for medicinal applications.

## Supplementary Material

Additional File 1 **Plate a**: Kusume women carrying *Moringa stenopetala *to Gato market place. The picture indicates that gathering and selling of wild plants is a common practice during food shortage. Click here for file

Additional File 2 **Plate b**: Cultivated and wild leafy edibles at market place. The illustration shows economic significance of wild plants to cash poor people. Click here for file

Additional File 3 **Plate c**: Fire setting (Gamo shrub land). The picture shows potential impact of fire on wild plant diversity. Click here for file

Additional File 4 **Plate d**: Damage after fire (Gamo shrub land). The picture shows potential impact of fire on wild plant diversity. Click here for file

Additional File 5 **Plate e**: Poles and timber sale at different market places of Derashe district. Selective harvesting of some exceptionally useful plant specie at market places. Click here for file

Additional File 6 **Plate e**: Poles and timber sale at different market places of Derashe district. Selective harvesting of some exceptionally useful plant specie at market places. Click here for file

Additional File 7 **Appendix 1 **List of wild edible plant species, habit, uses, parts used and habitat distribution. The list presents botanical and vernacular names of wild edible plants, growth forms, habitat distribution, additional uses and parts used. Click here for file
