# Supplementary figures and images for: Ethnobotanical study of wild edible plants in Derashe and Kucha Districts, South Ethiopia
Source: J Ethnobiol Ethnomed. 2006 Dec 21;2:53. doi: 10.1186/1746-4269-2-53 (PMC1769355; doi:10.1186/1746-4269-2-53)

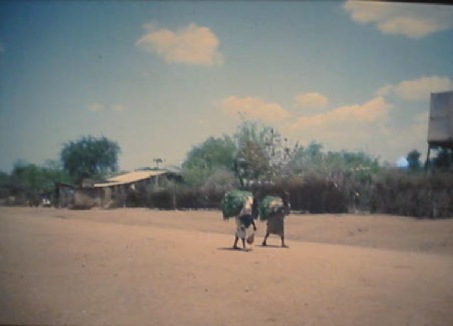

Supplement: Additional File 1 — Plate a: Kusume women carrying Moringa stenopetala to Gato market place. The picture indicates that gathering and selling of wild plants is a common practice during food shortage. [file 1746-4269-2-53-S1.JPEG]

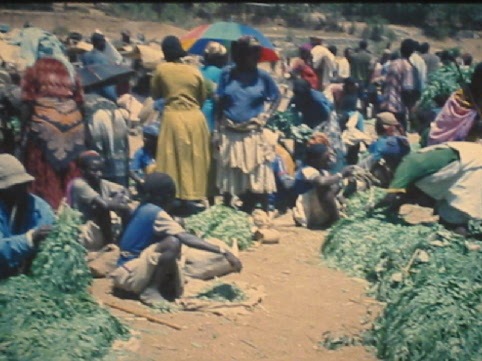

Supplement: Additional File 2 — Plate b: Cultivated and wild leafy edibles at market place. The illustration shows economic significance of wild plants to cash poor people. [file 1746-4269-2-53-S2.JPEG]

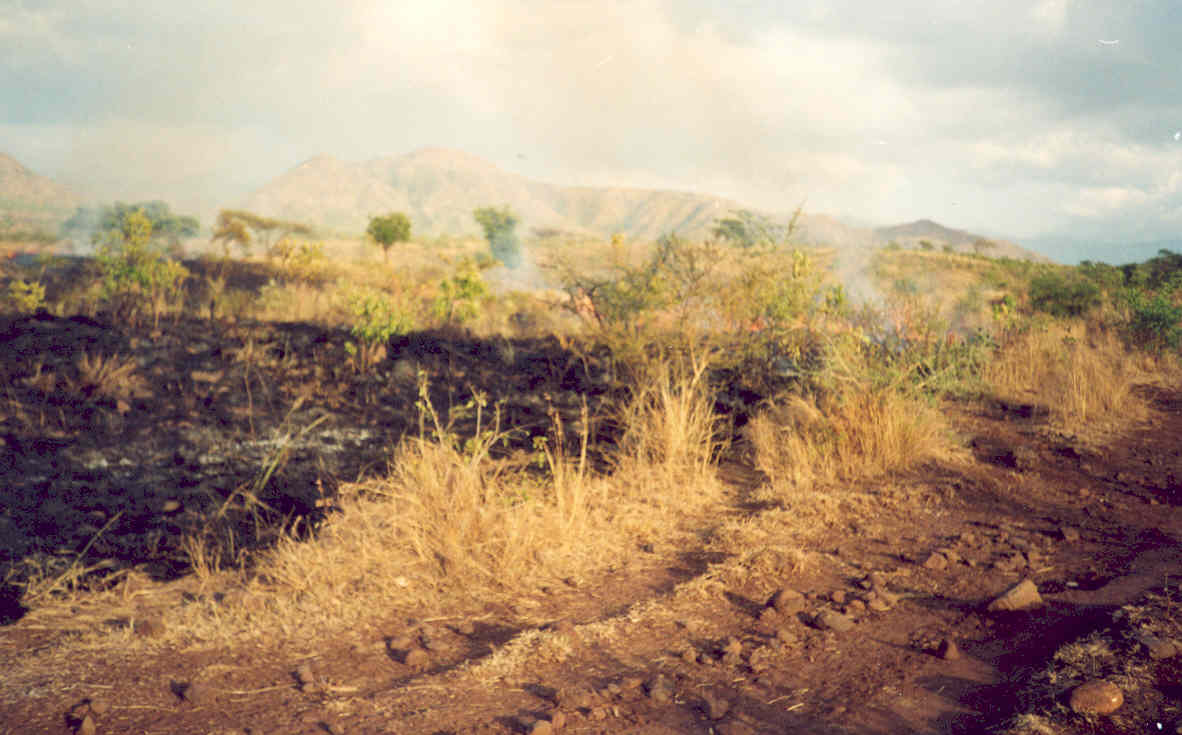

Supplement: Additional File 3 — Plate c: Fire setting (Gamo shrub land). The picture shows potential impact of fire on wild plant diversity. [file 1746-4269-2-53-S3.JPEG]

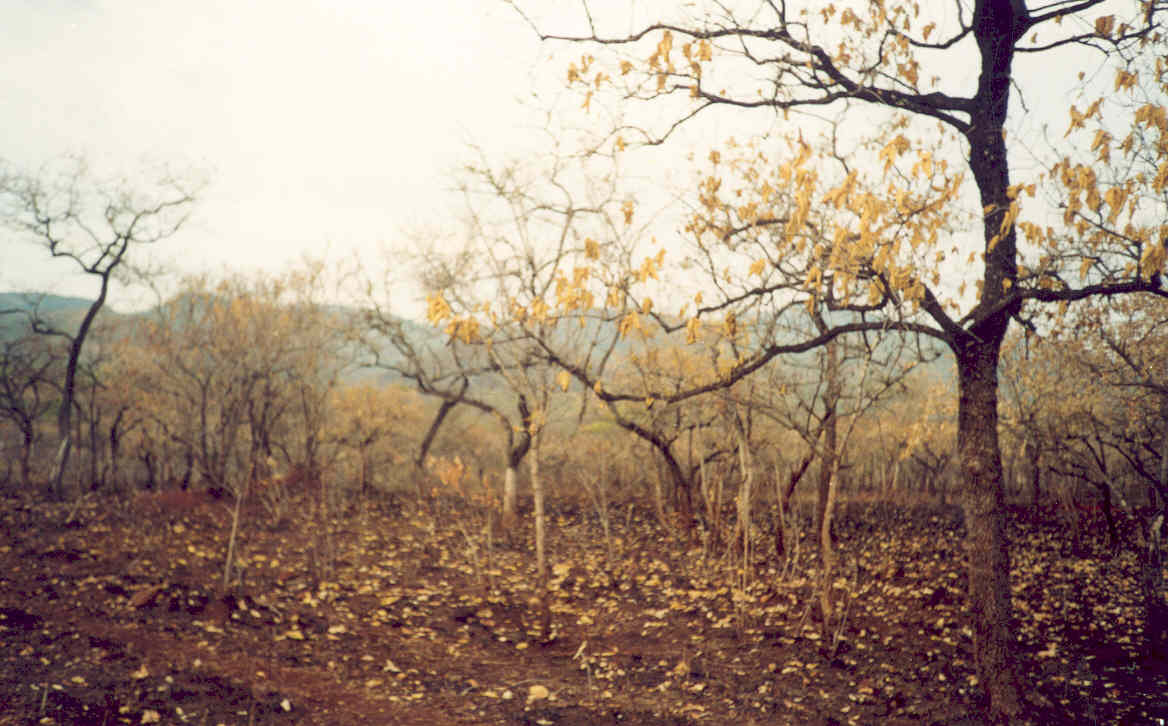

Supplement: Additional File 4 — Plate d: Damage after fire (Gamo shrub land). The picture shows potential impact of fire on wild plant diversity. [file 1746-4269-2-53-S4.JPEG]

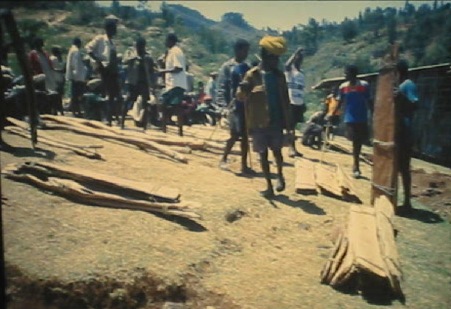

Supplement: Additional File 5 — Plate e: Poles and timber sale at different market places of Derashe district. Selective harvesting of some exceptionally useful plant specie at market places. [file 1746-4269-2-53-S5.JPEG]

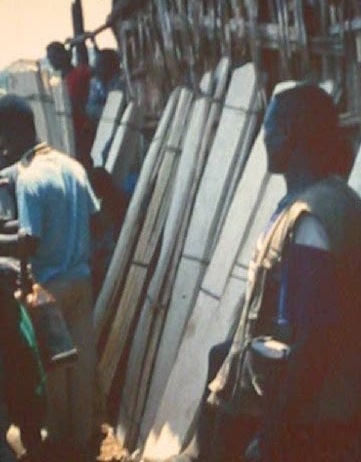

Supplement: Additional File 6 — Plate e: Poles and timber sale at different market places of Derashe district. Selective harvesting of some exceptionally useful plant specie at market places. [file 1746-4269-2-53-S6.JPEG]
