# Supplementary material for: Ethnobotanical study of wild edible plants in Derashe and Kucha Districts, South Ethiopia
Source: J Ethnobiol Ethnomed. 2006 Dec 21;2:53. doi: 10.1186/1746-4269-2-53 (PMC1769355; doi:10.1186/1746-4269-2-53)
Supplement: Additional File 7 — Appendix 1 List of wild edible plant species, habit, uses, parts used and habitat distribution. The list presents botanical and vernacular names of wild edible plants, growth forms, habitat distribution, additional uses and parts used. [file 1746-4269-2-53-S7.DOC]

Appendix 1 List of wild edible plant species, habit, uses, parts used and habitat distribution

| No | Scientific Names | Family | Vernacular names and Ethnic group  (K=Kusume; D=Derashe; G=Gamo) | Parts used | Habit | Habitat  Distribution | Other uses |
| --- | --- | --- | --- | --- | --- | --- | --- |
| 1 | *Acacia tortilis* (Forssk.) Hayne | Fabaceae | Dhetata (K, D) | B | Tree | Woodland | Fo; Fw |
| 2 | Amaranthus caudatus L. | Amaranthaceae | Gegebsa (G) | Se | Herb | Farmland | Me |
| 3 | *Amaranthus graecizans* L. | Amaranthaceae | Horoqota (D), Cumadhe (G) | YL | Herb | Farmland | _ |
| 4 | *Annona senegalensis* Pers. | Annonaceae | Monoqo (G) | F | Tree | Woodland | Co; Te |
| 5 | *Balanites aegyptiaca* (L.) Del. | Balanitaceae | Hangala (K), Hangalta (D), Domaye (G) | F, L | Tree | Woodland | Fo; Sh; Co; Te; Fi |
| 6 | *Balanites rotundifolia* (Van Tiegh.) Blatter | Balanitaceae | Kurarta (K), Busota/Pusota (D) | F | Shrub | Woodland | Me |
| 7 | *Bridelia micrantha* (Hochst.) Baill. | Euphorbiaceae | Xema (G) | Se | Shrub | Woodland | CO |
| 8 | *Capsicum annuum* L. | Solanaceae | Mixamixo (G) | F | Herb | River banks | _ |
| 9 | *Carissa spinarum* L. | Apocynaceae | Agamta (K), Agama (D), Lade (G) | F | Shrub | Woodland | Co; Te; Me |
| 10 | *Caylusea abyssinica* (Fresen.) Fisch. & Mey. | Resedaceae | Xomita (K, D) | L | Herb | Farmland | _ |
| 11 | *Celtis africana* Burm. f. | Ulmaceae | Dhawashya (K, D) | F | Tree | Woodland | Fo |
| 12 | *Clausena anisata* (Willd.) Benth. | Rutaceae | Funata (K, D) | F | Shrub | Woodland | Co; Fi |
| 13 | *Commelina diffusa* Burm. f. | Commellinaceae | Welilo (G) | YL | Herb | Disturbed land | _ |
| 14 | *Corchorus olitorius* L | Tiliaceae | Hololoqota (K, D); kepotugunta (G) | YL | Herb | Farmland | Fo; Me |
| 15 | *Corchorus trilocularis* L. | Tiliaceae | Shosha interse (G) | YL | Herb | Farmland | Fo |
| 16 | *Cordia africana* Lam | Boraginaceae | Otayita (K), Otaya (D), Meqota (G) | F | Tree | Woodland | Co; Te; Ri; Sh; Fi |
| 17 | *Cordia ovalis* R. Br. ex D.C. | Boraginaceae | Luketa (K, D) | F | Shrub | Woodland | Co; Te; Fi |
| 18 | Dioscorea praehensilis Benth. | Dioscoreacaeae | Welo (G) | R | Climber | River banks | _ |
| 19 | Diospyros abysinica (Hiern) F. White | Ebenaceae | Dul’o (G) | F | Tree | Woodland | Fi; Co; Te |
| 20 | *Dobera glabra* (Forssk.) Poir. | Salvadoraceae | Kerseta (K, D) | Se | Tree | Woodland | Sh, Hb |
| 21 | *Dombeya torrida* (G. F. Gamel) P. Bamps. | Sterculiaceae | Akota (K) | F | Tree | Woodland | Co; Fi; Ws |

**Appendix 1** Cont…

| No | Scientific Names | Family | Vernacular names and Ethnic group  (K=Kusume; D=Derashe; G=Gamo) | Parts used | Habit | Habitat  Distribution | Other uses |
| --- | --- | --- | --- | --- | --- | --- | --- |
| 22 | *Ehretia cymosa* Thonn. | Boraginaceae | Borborta (K), Maqaya (D), Itriwanje (G) | F | Tree | Woodland | Fo; Te |
| 23 | *Embelia schimperi* Vatke | Myrsinaceae | Inqoko (D) | F | Shrub | Forest | Me; Co, as tea |
| 24 | *Euclea divinorum* Hiern. | Ebenaceae | Dedeho (K) | Se | Shrub | Woodland | Co; Fi |
| 25 | *Ficus sycomorus* L. | Moraceae | Le’a (K), Hilteta (D), Eta (G) | F | Tree | Riverbank | Co; Te; Hb |
| 26 | *Ficus vasta* Forssk. | Moraceae | Artyita/Ardayida (K), Fofeya (D), Wela (G) | F | Tree | Riverbank | Co ; Te, Ri ; La ; Me ; Hb |
| 27 | *Flueggea leucopyrus* Willd. | Euphorbiaceae | Rarata (K), Hebata (D) | Se | Shrub | Woodland | Co; Fi |
| 28 | *Grewia bicolor* Juss. | Tiliaceae | Tsewayita (K) | F | Shrub | Woodland | Fi; Co; Ro; Ws |
| 29 | *Grewia* *mollis* Juss. | Tiliaceae | Tsewayita (G) | F | Shrub | Woodland | Fi; Co; Ro; Ws |
| 30 | *Grewia schweinfurthii* Burret | Tiliaceae | Qorawaqo (K) | F | Shrub | Woodland | Co; Fi |
| 31 | *Grewia villosa* Willd. | Tiliaceae | Luketa (K), Ogadie (G) | F | Shrub | Woodland | Co; Fi |
| 32 | Hypoestes forskaolii | Acanthaceae | Qirqirta/Qirqira (K), Ononayta (D) | L | Herb | Farmland | _ |
| 33 | *Lanatana rhodesiensis* Mold. | Verbenaceae | Untaorayitate (D) | Se, L | Shrub | Farmland | As tea/coffee |
| 34 | *Lepisanthes senegalensis* (Poir) Leenh. | Sapindaceae | Celle (G) | F | Tree | Woodland | Sh; Co; Fi |
| 35 | *Leptadenia hastata* (Pers.) Decne. | Asclepiadaceae | Haila (K, D) | L | Climb | Riverbank | Co |
| 36 | *Lucata* Spp. | Asteraceae | Maxo (G) | L | Herb | Farmland | _ |
| 37 | *Mussaenda arcuata* Poir. | Rubiaceae | Mirxako/ Mixaro(G) | F | Shrub | Woodland | Fi |
| 38 | *Mimusops kummel* Bruce ex A.DC. | Sapotaceae | Gurcho (G) | Se | Tree | Woodland | Sh; Fi; `Ri |
| 39 | *Myrsine africana* L. | Myrsinaceae | Xinqitata (D) | Se | Shrub | Woodland | Co |
| 40 | *Oncoba spinosa* Forssk. | Flacourtiaceae | Hagile (G) | F | Shrub | Woodland | Co; Fi |
| 41 | *Opuntia ficus-indica* (L.) Miller | Cactaceae | Holeta (K, D) | F | Herb | Farm field | Fo |
| 42 | *Pavetta abyssinica* Fresen. | Rubiaceae | Maduginata (K) | F | Shrub | Woodland | Fi |
| 43 | *Pentarrhinum inspidum* E. Mey. | Asclepiadaceae | Kokorpha (D) | L | Climber | Woodland | _ |
| 44 | *Phoenix reclinata* Jacq. | Arecaceae | Zamba (D) | F | Shrub | Forest margin | Ba |
| 45 | *Physalis peruviana* L. | Solanaceae | Awxetecha (D) | F | Herb | Farmland | _ |

Appendix 1 Cont…

| No | Scientific Names | Family | Vernacular names and Ethnic group  (K=Kusume; D=Derashe; G=Gamo) | Parts used | Habit | Habitat  Distribution | Other uses |
| --- | --- | --- | --- | --- | --- | --- | --- |
| 46 | *Piliostigma thonningii* (Schumach.)  Milne-Redh. | Fabaceae | Qalqala (G) | F | Tree | Woodland | Fi; Ro |
| 47 | *Portulaca quadrifida* L. | Portulacaceae | Mereqita (K), Meredheta (D), Mergude (G) | Ab | Herb | Farmland | Fo |
| 48 | *Rhus glutinosa* Hochst. | Anacardiaceae | Letata (D), Temo/Xemo (G) | Se | Shrub | Woodland | Co |
| 49 | *Rhus longipes* Engl. | Anacardiaceae | Ungafree (G) | Se | Shrub | Woodland | Co; Fi |
| 50 | *Rhus natalensis* Krauss | Anacardiaceae | Tomotayita (K, D) | F | Shrub | Woodland | Co; Fi; Fo |
| 51 | *Rhus ruspolii* Engl. | Anacardiaceae | Qacawuleteta (D), Mucako (G) | Se | Shrub | Woodland | Co; Te; Me; Fi |
| 52 | *Rubus apetalus* Poir. | Rosaceae | Komora (D), Injera (G) | F | Shrub | Forest | Co |
| 53 | *Saccharum spotaneum* L. | Poaceae | Maqesha (D) | St | Herb | River banks | _ |
| 54 | Sclerocarya birrea (A. Rich) Hochst. | Anacardiaceae | Baqusha/ba’ita (K), Pa’sha (D), Weslecha (G) | F | Shrub | Woodland | Co; Te; Fi |
| 55 | *Solanum nigrum* L. | Solanaceae | Qaqaha (K), Hundha (D), A’inaa comadhe (G) | F | Herb | Farmland | Me |
| 56 | *Sporobolus pyramidalis* P. Beauv. | Poacae | Girole (G) | Se | Herb | Disturbed land | Fo |
| 57 | Sterculia africana (Lour.) Fiori | Sterculiaceae | Qawrenta (K), Qawreta (D) | Se | Tree | Woodland | Co; Fi; Hb |
| 58 | *Strychnos innocua* Del. | Loganiaceae | Ugugee (G) | F | Shrub | Woodland | Co; Fi |
| 59 | *Syzygium guineense* (Willd.) Dc*.* | Myrtaceae | Kayinata (K, D), Ocha (G) | F | Tree | Forest | Co; Te; Fi |
| 60 | *Tamarindus indica* L. | Fabaceae | Kore (G) | F | Tree | Woodland | Sh; Ri; Fi |
| 61 | *Vanguieria apiculatum* K. Schum. | Rubiaceae | Gurmase (G) | F | Tree | Wood land | Co |
| 62 | *Ximenia americana* L. | Olacaceae | Sengigta (K), Mutul’eta (D), Unko/Mulaho (G) | F | Shrub | Woodland | Co; Fi; Me |
| 63 | *Ximenia caffra* L. | Olacaceae | Aste (G) | F | Shrub | Woodland | Co; Fi |
| 64 | *Zanthoxylum chalybeum* Engl. | Rutaceae | Ketata (K), Keta’ata (D), Dedeho (G) | Se, L | Tree | Woodland | Me, as tea/coffee |
| 65 | *Zizyphus mucronata* Willd | Rhamnaceae | Kobta (K) | F | Tree | Woodland | Co; Fi |
| 66 | *Zizyphus mauritiana* Lam | Rhamnaceae | Silka (G) | F | Tree | Woodland | Co; Te |

**Note** :-(Hb= Hanging beehives; Sh=Shade; Me=Medicinal; Fw=Fuel wood (Ch=Charcoal, Fi=Fire wood); Co=Construction; Te=Technology; Ri=Rituals; Fo=Forage; Ro=Rope; Ws=Walking stick; La=Latex; Ba=Basket
